# The Effect of Dexamethasone-Mediated Atrophy on Mitochondrial Function and BCAA Metabolism During Insulin Resistance in C2C12 Myotubes

**DOI:** 10.3390/metabo15050322

**Published:** 2025-05-13

**Authors:** Kayla J. Ragland, Kipton B. Travis, Emmalie R. Spry, Toheed Zaman, Pamela M. Lundin, Roger A. Vaughan

**Affiliations:** 1 Department of Health and Human Performance, High Point University, High Point, NC 27268, USA; kragland@highpoint.edu (K.J.R.); ktravis@highpoint.edu (K.B.T.); espry@highpoint.edu (E.R.S.); 2 Department of Chemistry, High Point University, High Point, NC 27268, USA; tzaman@highpoint.edu (T.Z.); plundin@highpoint.edu (P.M.L.)

**Keywords:** leucine, isoleucine, valine, sarcopenia, skeletal muscle, insulin resistance, diabetes

## Abstract

**Background**: Muscle loss during sarcopenia and atrophy is also commonly associated with age-related insulin resistance. Interestingly, branched-chain amino acids (BCAA) which are known for stimulating muscle protein synthesis are commonly elevated during insulin resistance and sarcopenic obesity. **Objectives**: This study investigated the effects of the interplay between atrophy and insulin resistance on insulin sensitivity, mitochondrial metabolism, and BCAA catabolic capacity in a myotube model of skeletal muscle insulin resistance. **Methods**: C2C12 myotubes were treated with dexamethasone to induce atrophy. Insulin resistance was induced via hyperinsulinemia. Gene and expression were measured using qRT-PCR and Western blot, while mitochondrial and lipid content were assessed using fluorescent staining. Cell metabolism was analyzed via Seahorse metabolic assays. **Results**: Both dexamethasone-induced atrophy and insulin resistance independently reduced insulin-stimulated pAkt levels, as well as mitochondrial function and content. However, neither treatment affected gene or protein expression associated with mitochondrial biogenesis or content. Although dexamethasone independently reduced insulin sensitivity in otherwise previously insulin-sensitive cells, dexamethasone had no significant effect on extracellular BCAA content. **Conclusions**: Our findings indicate the metabolic interplay between atrophy and insulin resistance and demonstrate that both can reduce mitochondrial function, though only limited effects were observed on indicators of BCAA catabolism and utilization. This emphasizes the need for future studies to investigate the mechanisms that underlie atrophy and other metabolic disorders to develop new interventions.

## 1. Introduction

Age-related diseases such as sarcopenia and atrophy which are often characterized by hallmark muscle loss, are complicated pathologies that are commonly associated with metabolic diseases and complications, including diabetes and insulin resistance [1]. A recent review highlights the interplay between the pathology of insulin resistance and sarcopenia, with emphasis on several well-studied molecular targets, including the mechanistic/mammalian target of rapamycin (mTOR) (a primary regulator of protein synthesis) and Akt (a central node in insulin signaling), among others [1]. The report also highlights the importance of skeletal muscle oxidative capacity and type I fiber abundance, which are often reduced in populations with insulin resistance and sarcopenia [1]. Interestingly, within the interplay between insulin resistance and sarcopenia, the branched-chain amino acids (BCAA) have emerged as important metabolites [1].

For example, BCAA appear to consistently be elevated with increasing insulin resistance [2,3,4,5,6,7,8,9]. Along with BCAA accumulation, elevated concentrations of the valine catabolite 3-hydroxyisobutyrate (3-HIB) has also been observed in subjects with insulin resistance [10,11,12]. Collectively, the downregulation of BCAA catabolic enzymes, branched-chain alpha-keto acid dehydrogenase (BCKDH) and branched-chain aminotransferase (BCAT), has been implicated as a contributing factor [13]. Additionally, mitochondrial dysfunction as a result of BCAA accumulation has also been proposed as a contributing mechanism by which BCAA may promote insulin resistance [5,6]; however, evidence also exists demonstrating BCAA improve mitochondrial function [14,15,16,17].

Conversely, there are inconsistencies on the effects of BCAA during age-related muscle loss. While studies investigating the effect of protein/BCAA-rich diets have largely beneficial effects on preservation of muscle content and function [1], some populations suffering from sarcopenic obesity may exhibit a seemingly paradoxical constellation of elevated BCAA with reduced muscle mass, muscle function, and insulin sensitivity [18]. In part, this may be resultant from the confounding effects of insulin resistance during muscle loss, which may alter the way BCAA are metabolized. Evidence for such effects might be illustrated by comparing healthy obesity versus unhealthy obesity, such as that performed by Petersen et al., who demonstrated important metabolic disparities in the skeletal muscle of healthy and unhealthy obese subjects [19]. Of great interest was the observation that those with unhealthy obesity exhibited reduced expression of BCAA catabolic enzymes and reduced mitochondrial function, which corresponded with significantly elevated circulating BCAA [19]. Several in vitro reports have demonstrated an effect of the atrophy induction of dexamethasone to reduce muscle mitochondrial content and function [20,21,22,23], as well as insulin sensitivity [20,24,25,26,27]. Limited evidence also suggests myotube atrophy induced by dexamethasone may diminish myotube L-type amino acid transporter 1 (LAT1) [28], the predominant transporter of BCAA in skeletal muscle; however, it is important to note other transporters appear to compensate for reduced LAT1 expression and BCAA uptake [29,30,31].

Together, past evidence has demonstrated a link between muscle loss (such as that seen in sarcopenia), insulin resistance, and the accumulation of metabolites such as BCAA in circulation due to reduced BCAA metabolism. However, while past evidence has revealed this link, to our knowledge, no study has thoroughly investigated the effects of muscle loss (such as that seen during atrophy and/or sarcopenia), both in the absence and presence of insulin resistance, on skeletal muscle metabolism, including BCAA metabolism and utilization. Therefore, the purpose of the current report was to investigate the effects of mild and severe dexamethasone-induced myotube atrophy (as a surrogate for atrophy) on insulin sensitivity, mitochondrial metabolism, and BCAA disposal and catabolic capacity. It is worth acknowledging that there are several limitations of the myotube model and the limited generalizability to human pathologies such as atrophy and sarcopenia. In general, it is anticipated that atrophy will reduce myotube mitochondrial function, as will insulin resistance. It is also anticipated that the co-conditioning of cells with both insulin resistance and atrophy will lead to a further reduction in insulin sensitivity, mitochondrial function, and BCAA utilization. Below, we detail our efforts to quantify the various possible effects of dexamethasone on both insulin-sensitive and insulin-resistant cells to parse out the relationships of insulin sensitivity, mitochondrial function, and BCAA metabolism.

## 2. Experimental Methods

### 2.1. Cell Culture

C2C12 mouse myoblasts from ATCC (Manassas, VA, USA) were cultured and differentiated under standard conditions as previously described (using cell passages <20 for all experiments) [32]. Insulin resistance was accomplished by the addition of insulin at 100 nM for the final 3 days of differentiation as previously performed [33,34,35,36,37]. Similar to previous investigations [24,25,26,28], atrophy was induced by treating cells with either 1 µM or 10 µM dexamethasone (Santa Cruz Biotechnology, Dallas, TX, USA) or DMSO control (final volume of DMSO was 0.1% vol:vol for all groups) for 24 h. These concentrations of dexamethasone were selected as sufficient levels to induce atrophy [24,25,26,28], and because previous data in hepatocytes suggests that similar levels of dexamethasone alter BCKDH activity [38].

### 2.2. Quantitative Real Time Polymerase Chain Reaction (qRT-PCR)

Following treatment as described above, mRNA was extracted using the Trizol method, and cDNA was synthesized using the iScript cDNA Synthesis Kit from Bio-Rad (Hercules, CA, USA). PCR primers were synthesized by Integrated DNA Technologies (Coralville, IA, USA) (Table S1), and target genes were normalized to the housekeeping gene TATA binding protein (Tbp), which did not differ between groups (Figure S1). qRT-PCR was performed using SYBR Green-based reactions and cycling parameters as previously described [32]. qRT-PCR reactions were performed using *n* = 2 per treatment condition from two independent experiments, with *n* = 4 for the final analysis. Relative quantification was determined via the ΔΔCt method.

### 2.3. Immunoblotting

Cells were differentiated and treated as described, and collected on ice in RIPA. To assess insulin sensitivity, cells were treated as described above, followed by serum-free media stimulation with 100 nM insulin for 30 min. Samples were size-separated, transferred to PVDF, and blocked in TBST-5% non-fat milk powder. Membranes were probed overnight with primary antibodies at 4 °C in TBST-5% non-fat milk powder, followed by rinsing and secondary antibody exposure for 1 h at room temperature (Table S2). Signal intensities were determined by chemiluminescence and quantified using Image Lab from Bio-Rad (Hercules, CA, USA). Blots were performed using two replicates per condition, across two independent experiments, with *n* = 4 for the final analysis.

### 2.4. Seahorse Metabolic Assays

As previously performed [32], cells were subjected to a MitoStress test following 24-h treatment as described above. Cells were sequentially exposed to oligomycin, carbonyl cyanide p-[trifluoromethoxy]-phenyl-hydrazone (FCCP), and rotenone, and measurements were recorded following each exposure. Each MitoStress experiment included *n* = 15–16 per group, repeated with two independent experiments for a total of *n* = 30–32 per group for the final analysis. States of mitochondrial metabolism were calculated by subtracting non-mitochondrial respiration from basal or FCCP-induced peak mitochondrial oxygen consumption. Wells with negative OCR values or no response to injection were removed from the final analysis. Data was normalized to relative nuclei content (Figure S2).

### 2.5. Fluorescent Staining and Fluorescent Microscopy

Following the Seahorse MitoStress experiments, cells were fixed in media containing 3.7% formaldehyde at 37 °C with a 5% CO_2_ atmosphere. Cells were then sequentially stained with DAPI, then rinsed and stained with nonyl acridine orange (NAO), and finally rinsed and stained with Nile Red as previously performed, and fluorescents for each measured as previously described [32]. Myotube fusion index and diameter were assessed using phase and DAPI-stained images of myotubes using the 20× objective. Images were quantified by a blinded member of the research team.

### 2.6. Liquid Chromatography–Mass Spectrometry (LC–MS)

Extracellular BCAA accumulation was assessed by measuring conditioned media following treatment as described above. Measurements were performed via LC/MS using a Shimadzu Nexera UHPLC system equipped with a Phenomenex Kinetex C18 100Å column using conditions previously reported [39]. Stock BCAA diluted in a water/methanol solution were used to create standards used to determine the concentration of each BCAA. Shimadzu LabSolution software version 5.97 was used to acquire and process the data. Experiments were performed using four replicates per group for each of two independent experiments, with *n* = 8 for each group in the final analysis, with each sample measured in triplicate.

### 2.7. Statistical Analyses

Data are presented as dot plots with group means or as group mean ± SE. Seahorse MitoStress time trial data were analyzed using factorial ANOVA with time as a repeated measures factor and Bonferroni’s correction for pairwise group differences. All other data were analyzed with two-way ANOVA with Bonferroni’s correction for pairwise group differences. Data were also assessed for normality using Kolmogorov-Smirnov’s test, and data sets with non-normal data were analyzed by nonparametric post-hoc comparisons using Dunn’s multiple comparison test. In cases of suspected extreme outliers, Grubb’s test was performed prior to the removal of any verified outlier. Values of *p* < 0.05 were used to identify significant differences between groups.

### 2.8. Additional Materials, Methods, and Experimental Procedures

Please see the supplemental materials for additional details for each experimental resource and procedure. Details for qRT-PCR primers and primary/secondary antibodies are also listed in supplemental materials within Table S1 and Table S2, respectively.

## 3. Results

### 3.1. Dexamethasone-Induced Atrophy Reduces pAKT Expression in Cultured Myotubes

**Figure 1:**
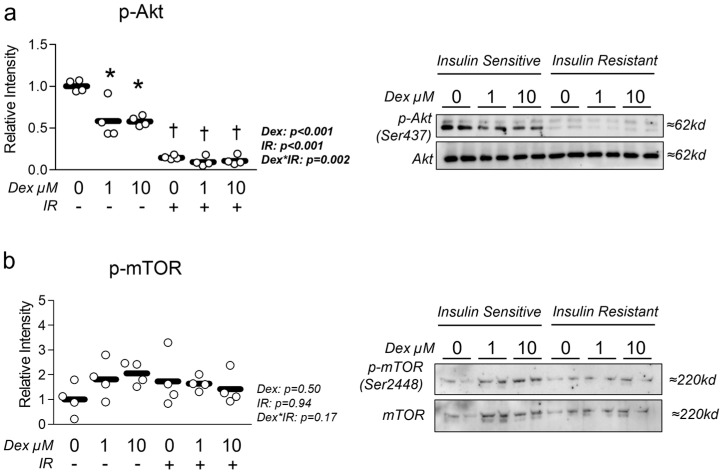
Effect of dexamethasone on insulin sensitivity. (**a**,**b**) Effect of dexamethasone (Dex) at 0 µM, 1 µM, or 10 µM or DMSO control (0.1%) for 24 h under insulin-sensitive or insulin-resistant (IR) conditions on (**a**) p-Akt and (**b**) p-mTOR activation following insulin stimulation for 30 min. Notes: Two-way ANOVA with Bonferroni’s corrections was used to analyze data. *p* values presented for main effects are listed for each outcome. * Indicates *p* < 0.05 between Dex versus its true control within each level of insulin sensitivity. † Indicates *p* < 0.05 between levels of insulin resistance within similar Dex concentrations. Data are from two replicate wells from two independent experiments (*n* = 4).

We began our experiments by verifying reduced insulin-mediated Akt activation in insulin-resistant cells and assessing the effect of dexamethasone-induced atrophy on insulin signaling. As anticipated, p-Akt activity indicated by phosphorylation status was significantly depressed in insulin-resistant cells (Figure 1a). In addition, Dex-only treatment also resulted in a significant reduction in p-Akt expression, suggesting Dex independently promotes insulin resistance (Figure 1a). Next, we assessed the effect of each treatment condition on mTOR activation and found no effect of either condition on p-mTOR activity indicated by phosphorylation status normalized to total mTOR abundance (Figure 1b).

### 3.2. Dexamethasone Reduces Myotube Diameter and Fusion Index, Which Is Unaffected by Insulin Resistance

**Figure 2:**
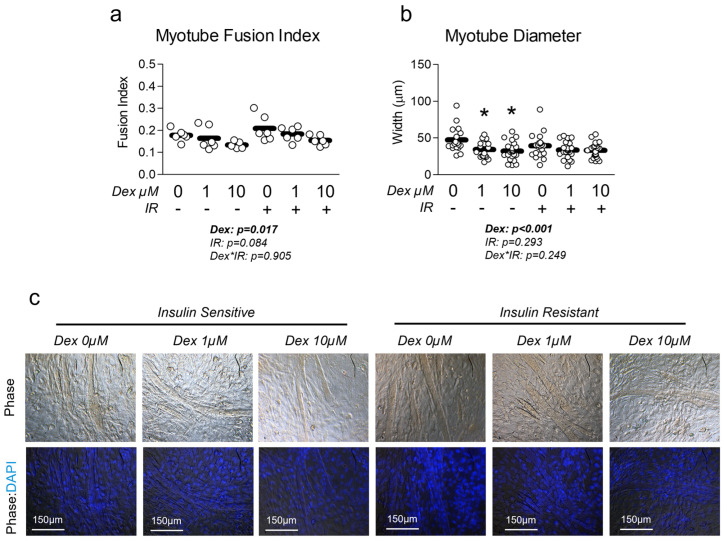
Effect of dexamethasone and insulin resistance on myotube fusion and diameter. (**a**,**b**) Effect of dexamethasone (Dex) at 0 µM, 1 µM, or 10 µM or DMSO control (0.1%) for 24 h under insulin-sensitive or insulin-resistant (IR) conditions on (**a**) myotube fusion index and (**b**) myotube diameter. Notes: Two-way ANOVA with Bonferroni’s corrections was used to analyze data. *p* values presented for main effects are listed for each outcome. * Indicates *p* < 0.05 between Dex versus its true control within each level of insulin sensitivity. No differences were observed between levels of insulin resistance within similar Dex concentrations. Myotube fusion was calculated from three replicate wells from two independent experiments (*n* = 6) and was quantified by a blinded member of the research team with representative images of phase and DAPI-phase overlay depicted in panel (**c**). One value within the insulin-sensitive Dex 10 µM group was removed from the final analysis as a significant outlier (*p* < 0.05, Grubb’s outlier test). Myotube diameter was calculated using images from three replicate wells from two independent experiments, with individual myotubes assessed for diameter by a blinded member of the research team (*n* = 17–24 myotubes per group).

Next, we sought to measure the effect of dexamethasone treatment on myotube phenotype in the presence and absence of insulin resistance. Cells treated with dexamethasone displayed significantly reduced myotube fusion index (Figure 2a). This induction of atrophy was further confirmed by the reduced myotube diameter in cells treated with dexamethasone (Figure 2b). To our surprise, neither treatment had any significant effect on common atrophy-related targets of MuRF1 or Atrogin-1 mRNA expression (Figure S3), however, we did observe a significant reduction in total FOXO1 at the protein (but not mRNA) level (Figure S4), which is a known regulator of MuRF1 and Atrogin1. We also assessed the effect of each treatment condition on myosin heavy chain expression and found no difference in Myh7 expression, which is expressed in more oxidative, type I fibers (Figure S5). However, we did observe a significant increase in Myh1 expression, which is associated with a more glycolytic phenotype (Figure S5).

### 3.3. Dexamethasone-Induced Atrophy Reduces Myotube Mitochondrial Content and Function, Which Is Worsened by Insulin Resistance

**Figure 3:**
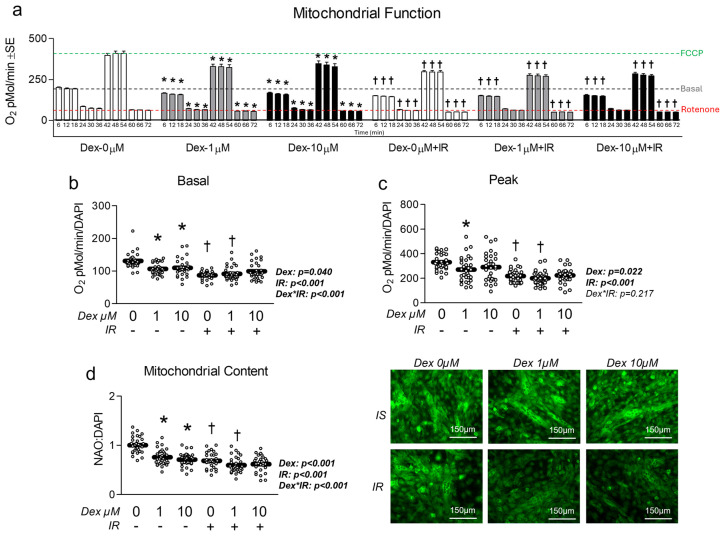
Effect of dexamethasone on mitochondrial function. (**a**) MitoStress assay following treatment with dexamethasone (Dex) at 0 µM, 1 µM, or 10 µM or DMSO control (0.1%) for 24 h under insulin-sensitive or insulin-resistant (IR) conditions. (**b**,**c**) Effect of Dex on basal (**b**) and peak (**c**) mitochondrial metabolism following normalization to cell nuclei content (presented in Figure S2). (**d**) Mitochondrial content (NAO staining) of cells treated as described in “a” following normalization to cell nuclei content (presented in Figure S2). Notes: Panel “a” was analyzed using factorial ANOVA with time as a repeated measures factor and Bonferroni’s correction for multiple comparisons. Two-way ANOVA with Bonferroni’s corrections was used to analyze data. *p* values presented for main effects are listed for each outcome. * Indicates *p* < 0.05 between Dex versus its true control within each level of insulin sensitivity. † Indicates *p* < 0.05 between levels of insulin resistance within similar Dex concentrations. Images presented in panel “d” were captured using the 20× objective. Data are from 15 to 16 replicate wells from two independent experiments (*n* = 30–32).

**Figure 4:**
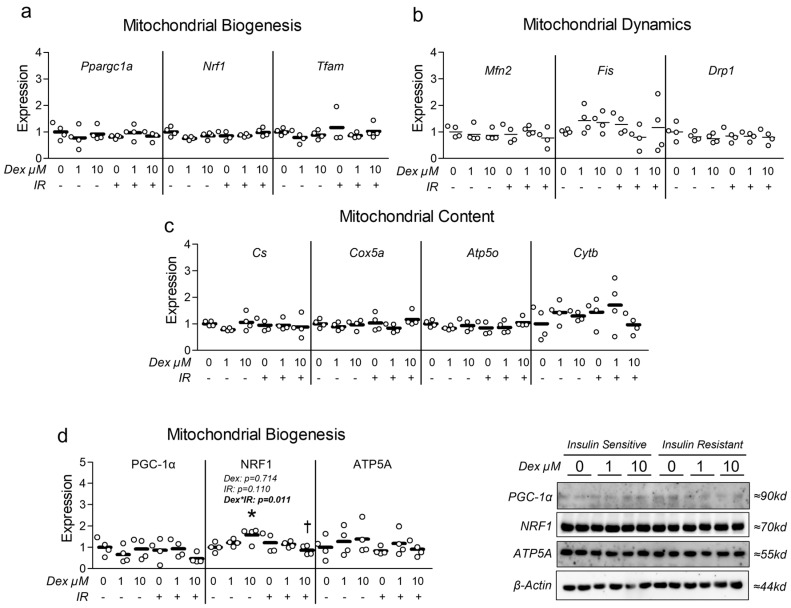
Effect of dexamethasone on mitochondrial biogenesis and dynamics. (**a**–**c**) Effect of dexamethasone (Dex) at 0 µM, 1 µM, or 10 µM or DMSO control (0.1%) for 24 h under insulin-sensitive or insulin-resistant (IR) conditions on mRNA expression of (**a**) mitochondrial biogenesis, (**b**) mitochondrial fusion and fission (dynamics), and (**c**) respiratory components (content). (**d**) Effect of treatment as described in “a” on protein expression of regulators of mitochondrial biogenesis and mitochondrial content. Notes: Two-way ANOVA with Bonferroni’s corrections was used to analyze data. *p* values presented for main effects are listed for each outcome when significant main/interaction effects were observed. Gene expression results revealed no significant effects. * Indicates *p* < 0.05 between Dex versus its true control within each level of insulin sensitivity. † Indicates *p* < 0.05 between levels of insulin resistance within similar Dex concentrations. Data are from two replicate wells from two independent experiments (*n* = 4).

Given the association between insulin resistance and reduced mitochondrial function, we assessed the effect of Dex in the presence and absence of insulin resistance on myotube mitochondrial function. Congruent with this idea, cells treated with Dex and/or insulin resistance exhibited significantly reduced oxygen consumption in a dose-dependent manner (Figure 3a), indicating reduced mitochondrial function. Moreover, following removal of non-mitochondrial respiration and normalization to nuclei content, basal mitochondrial oxygen consumption was significantly reduced by both Dex and insulin resistance, which is suggestive of reduced basal mitochondrial respiration (Figure 3b). Interestingly, insulin-resistant cells treated with 1 µM Dex displayed a further reduction in basal mitochondrial oxygen consumption than insulin-sensitive cells treated with 1 µM Dex (Figure 3b), which may suggest an additive effect of insulin resistance when coupled with dexamethasone-induced myotube atrophy (interaction effect *p* < 0.001). Peak mitochondrial oxygen consumption was also significantly reduced following either Dex treatment or insulin resistance independently, however, no additive effect was observed when the two treatments were coupled (Figure 3c). To determine if mitochondrial content was altered in proportion to mitochondrial function, we compared mitochondrial staining between groups and again found both Dex and insulin resistance independently reduced mitochondrial content, and that insulin-resistant cells treated with 1 µM Dex displayed a further reduction in mitochondrial content than insulin-sensitive cells treated with 1 µM Dex (Figure 3d). Collectively, these observations demonstrate that each treatment condition is sufficient to reduce myotube mitochondrial function and content. It is, however, noteworthy that there was no difference between the insulin-resistant cells treated with 10 µM Dex versus the insulin-resistant-only control or the insulin-sensitive cells treated with 10 µM Dex for any of the assessed mitochondrial function or content experiments. This may imply that the 10 µM is sufficient to diminish mitochondrial function to a baseline level. To explore the potential mechanisms of Dex and insulin resistance-mediated reductions in mitochondrial function/content, we explored gene and protein expression of molecular targets that govern mitochondrial content and observed no major effect of either treatment condition on molecular regulators of mitochondrial biogenesis or dynamics (Figure 4). Because we did not observe any consistent changes in mitochondrial biogenesis or dynamics, we also assessed the effects of each treatment on the expression of Parkin at the mRNA and protein levels. Again, no significant changes between groups were observed (Figure S6).

### 3.4. Dexamethasone Does Not Alter BCAA Metabolism

**Figure 5:**
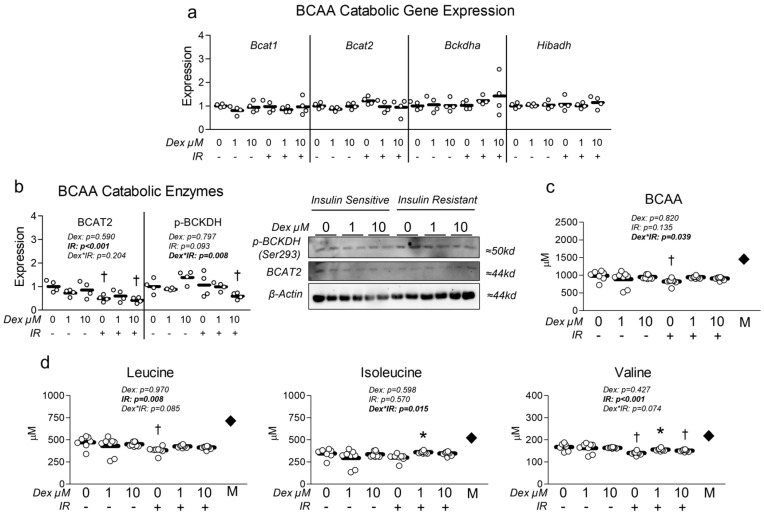
Effect of dexamethasone on branched-chain amino acid metabolism. (**a**) Effect of treatment with dexamethasone (Dex) at 0 µM, 1 µM, or 10 µM or DMSO control (0.1%) for 24 h under insulin-sensitive or insulin-resistant (IR) conditions on BCAA catabolic enzyme mRNA expression signaling. (**b**) Effect of treatment as described in “a” on BCAA catabolic enzyme protein expression signaling. (**c**,**d**) Effect of treatment as described in “a” on extracellular (**c**) total BCAA and (**d**) individual BCAA content. Notes: Two-way ANOVA with Bonferroni’s corrections was used to analyze data. *p* values presented for main effects are listed for each outcome when significant main/interaction effects were observed. Gene expression results revealed no significant effects. * Indicates *p* < 0.05 between Dex versus its true control within each level of insulin sensitivity. † Indicates *p* < 0.05 between levels of insulin resistance within similar Dex concentrations. mRNA and protein expression data are from two replicate wells from two independent experiments (*n* = 4). Media BCAA content was from four replicate wells from two independent experiments (*n* = 7–8). The dark diamond labeled “M” within panels (**c**,**d**) denotes stock media BCAA composition. One value within the insulin-sensitive control group was removed from the final analysis as a result of being a significant outlier (*p* < 0.05, Grubb’s outlier test).

Because both Dex and insulin resistance reduced mitochondrial metabolism and content, and because both muscle wasting disorders and insulin resistance correlate with dysregulation of BCAA metabolism, we assessed the effect of each condition on expression of primary BCAA catabolic enzymes (several of which function within the mitochondria). At the mRNA level, no effect of either treatment condition was observed (Figure 5a). However, at the protein level, insulin resistance significantly reduced BCAT2 expression (Figure 5b), the initial step in BCAA metabolism. We also observed an interaction effect between insulin resistance and Dex, during which insulin-resistant cells co-treated with Dex at 10 µM exhibited significantly reduced BCKDH phosphorylation (Figure 5b), which is indicative of increased BCKDH activity. Lastly, we investigated the effect of Dex on extracellular BCAA content and observed a significant interaction between insulin resistance and Dex treatment (Figure 5c). Analysis of individual BCAA revealed a main effect of insulin resistance in lowering extracellular leucine and valine (Figure 5d). Further group comparisons revealed that insulin resistance alone reduced extracellular leucine and valine, which was not consistently observed in insulin-resistant cells that also received Dex (Figure 5d). These data may suggest Dex may reduce BCAA metabolism, utilization, and/or uptake during existing insulin resistance.

## 4. Discussion

Muscle loss is associated with numerous diseases such as sarcopenia and atrophy, which are complicated pathologies that often occur with other co-morbidities including insulin resistance [1]. Interestingly, altered BCAA metabolism appears to be linked with both muscle loss [18] and insulin resistance [2,3,4,5,6,7,8,9]. In the present report, we sought to assess the effect of dexamethasone-induced myotube dysfunction as a model of atrophy on several aspects of metabolism, such as mitochondrial function, which is known to be reduced during both atrophy and insulin resistance. In the context of atrophy, mitochondrial dysfunction is specifically believed to be a primary contributing factor [40]. In fact, similar experiments performed using C2C12 myotubes treated with dexamethasone showed reduced mitochondrial function and ATP production [41]. Additionally, the same report demonstrated that muscle atrophy in the tibialis anterior of mice treated with dexamethasone is preceded by reductions in mitochondrial function [41]. Lui et al. concluded that the reductions in mitochondrial function and ATP levels precede the atrophic conditions and promote atrophic signaling in a positive feedback manner [41]. In the context of the present report, we observed consistently reduced mitochondrial function and reduced myotube size, but did not observe substantial upregulation of atrophic signaling. This disparity may be the result of insufficient treatment duration, or that changes in protein expression of atrophy-related targets were altered (but were not assessed by the current report). Additionally, we did not observe any significant alterations in mitochondrial biogenic or dynamic signaling despite consistent reductions in mitochondrial content and function. This is in opposition to our original hypothesis and several other observations demonstrating that dexamethasone-induced myotube atrophy results in reduced expression of key targets that regulate mitochondrial content [20,21,22]. Specifically, similar experiments using C2C12 myotubes treated with dexamethasone at 10 µM for 24 h showed reduced SIRT1, NRF1, and TFAM expression with increased acetylated PGC-1α expression (indicative of reduced mitochondrial biogenic signaling) [20]. Comparable findings in C2C12 cells also treated with 10 µM dexamethasone for 24 h showed reduced Ppargc1a, Nrf1, and Tfam mRNA expression [24]. Higher concentrations have also been assessed and showed that dexamethasone at 100 µM for 24 h also reduced PGC-1α expression [42]. While we did not observe this effect on mitochondrial biogenesis, we did consistently observe reduced mitochondrial function and staining, which may suggest our experiments were not optimized to capture this effect. Moreover, it is possible that other pathways associated with mitochondrial turnover and mitophagy are involved in reducing mitochondrial content during the tested treatment conditions, which warrants additional experiments. Collectively, our observations of reduced mitochondrial function and content are in line with several past observations which have also demonstrated reduced mitochondrial function and/or content in C2C12 myotubes treated with dexamethasone [20,21,22]. Biologically, these observations are important as they further implicate mitochondrial dysfunction in the development of both atrophy and insulin resistance.

Regarding insulin resistance, we directly assessed myotube response to insulin stimulation using pAkt expression following each treatment condition. We found that dexamethasone alone reduced insulin sensitivity in otherwise insulin-sensitive cells. This is in line with past observations in similar models that demonstrated dexamethasone reduced pAkt expression [20,24,25,26,27]. However, in cells with prior insulin resistance, dexamethasone did not cause a further reduction in insulin sensitivity (possibly because levels had been reduced to a basal level). This finding is contrary to our original hypothesis that dexamethasone-induced myotube atrophy would additively reduce insulin signaling when coupled with concurrent insulin resistance. However, the observation that cells treated with dexamethasone alone exhibited statistically lower insulin signaling and reduced mitochondrial function provides additional evidence linking the development of insulin resistance with mitochondrial dysfunction. We also investigated the effect of dexamethasone on glycolytic metabolism under both basal and oligomycin-induced peak glycolytic conditions (Figure S6a) and observed an increased reliance on glycolytic metabolism under basal conditions in dexamethasone-treated cells (Figure S7b). Interestingly, dexamethasone-treated cells retained their peak glycolytic capacity versus their corresponding insulin-sensitive or insulin-resistant controls (Figure S7c). Conversely, insulin-resistant cells displayed significantly reduced peak glycolytic metabolism, suggesting reduced overall glycolytic capacity (Figure S7c). Surprisingly, these reductions in glycolytic metabolism in insulin-resistant cells occurred without substantial changes in glycolytic gene expression (Figure S7d). Together, the observation that both insulin resistance and atrophy reduced mitochondrial function and glycolytic metabolism is evidence that each condition can independently reduce myotube metabolism.

Lastly, because BCAA can contribute to the regulation of muscle physiology and metabolism, the interplay between BCAA metabolism/abundance and muscle physiology may have several implications for muscle health. A recent paper by Chang et al. found reduced intramuscular BCAA content of sarcopenic mice with increased levels of circulating BCAA [43]. The same report found reduced LAT1 and BCAT2 abundance in the skeletal muscle of aged mice [43]. Mechanistically, LAT1 knockdown resulted in similar suppression of BCAT2 expression in cultured myotubes [43]. Chang et al. also demonstrated that induction of a sarcopenic phenotype using either palmitate or H_2_O_2_ resulted in reduced LAT1 expression [43]. The report further showed that palmitate reduced anabolic signaling including p-mTOR and downstream targets [43]. Thus, it is possible that LAT1-mediated alterations during muscle pathology, such as atrophy and sarcopenia, result in part from altered BCAA uptake and utilization [43]. In our experiments, neither dexamethasone nor insulin resistance altered gene expression of BCAA-catabolic targets during any of the tested conditions. However, we observed altered protein expression of BCAT2 and p-BCKDHa. This disparity in expression at the mRNA and protein levels might be explained by protein turnover or the intrinsic difference in the timing of changes in mRNA versus protein expression. The reduction in BCAT2 protein expression in insulin-resistant cells is in line with the observations by Chang et al. [43]. And as noted by Chang et al. [43], it has previously been discussed how reduced BCAT2 could result in increased protein turnover [8,44]. Unfortunately, however, findings from our study and Chang et al. are not perfectly comparable, as Chang used a different stimulus of muscle pathology (palmitate), and we unfortunately did not assess the effect of either treatment condition on LAT1 expression. That said, we investigated the effects of the dexamethasone model of muscle atrophy on BCAA utilization and found that Dex restored extracellular BCAA to control levels, which were otherwise reduced in insulin-resistant cells not treated with Dex. These data may suggest Dex might alter BCAA metabolism, utilization, and/or uptake, or a combination thereof. However, a limitation of our experiments was the use of only stock media BCAA concentrations, which may also contribute to the response of cells to the dexamethasone model. Additionally, we were unable to measure the alpha-ketoic acid metabolites of each BCAA, which limits the conclusions we can draw from extracellular BCAA abundance. Such data would add clarity to the potential metabolic fate of each BCAA (metabolism, storage, etc.). These limitations aside, the importance of skeletal muscle BCAA use in the context of metabolic disease and health has been previously identified and is worthy of additional experimentation [19]. Moreover, it appears that adequate protein and/or sufficient amino acids may provide some functional benefits in sarcopenia (though the findings are varied and warrant further investigation) [45].

Collectively, the present study provides foundational data on the interplay between dexamethasone-mediated atrophy and insulin resistance in a myotube model, and consistently demonstrates the suppressive effect of each condition on mitochondrial function. However, our report is not without other limitations. The myotube model itself is perhaps the largest limitation of the current report and is a limitation that seems ubiquitous to in vitro strategies used to investigate basic aspects of complex human physiology and diseases, such as atrophy and sarcopenia. Indeed, within the literature, C2C12 myotubes treated with dexamethasone are commonly used to emulate both atrophy and sarcopenia, though this strategy has numerous limitations and requires several assumptions and oversimplification of each disease. Additionally, given the dynamic nature of skeletal muscle, it would be worth examining similar models with varied BCAA availability (versus stock culture media BCAA composition only), such as those performed by Chang et al. [43]. Moreover, though we assessed extracellular BCAA levels, it is difficult to distinguish the fate of amino acids without numerous tracer experiments. Additionally, other amino acids may be altered during metabolic pathology, which warrants the need for investigations to also consider the abundance of other amino acids (namely glutamine/glutamate). And while our experiments were based on similar in vitro reports, it would be worth expanding the investigation beyond a single time point (though, importantly, others have demonstrated a reliable effect of dexamethasone treatment following 24-h treatment at the tested concentrations). These limitations aside, the present report demonstrates the ability of atrophy to reduce mitochondrial function which can be increasingly pronounced when coupled with insulin resistance. Yet, the exact effect of atrophy during insulin resistance on skeletal muscle metabolism remains a bit unclear and warrants further investigation.

## 5. Conclusions

The combination of insulin resistance, muscle loss, and circulating metabolites such as BCAA are complex. Our investigation assessed the effect of varied levels of dexamethasone-mediated atrophy on aspects of myotube metabolism, insulin sensitivity, and BCAA metabolism. It appears that dexamethasone-mediated atrophy consistently reduced mitochondrial function and promoted insulin resistance, independent of the hyperinsulinemic model used to induce insulin resistance as a separate treatment condition. Interestingly, dexamethasone-mediated atrophy did not induce a prominent effect on BCAA utilization (at least in the tested in vitro model). Future investigations should consider BCAA as a potentially meaningful target in the development and/or progression of atrophy, and consider the inherent limitations of the experimental model with the dynamic nature of amino acids in mind. This seems of special importance in the in vitro assessment of potential therapies using the proof-of-concept myotube model of atrophy/sarcopenia. Additional research will be needed to further assess other models of atrophy/sarcopenia to determine the relationship between insulin resistance, BCAA utilization, and loss of skeletal muscle.

## Data Availability

The data that support the findings of this study are presented within the manuscript and supplemental materials, and additional information is available from the corresponding author upon reasonable request.

## Supplementary Materials

The following supporting information can be downloaded at: https://www.mdpi.com/article/10.3390/metabo15050322/s1, Figure S1: qRT-PCR loading control; Figure S2: Nuclei content from Seahorse metabolic assays; Figure S3: Effect of dexamethasone model on atrophic mRNA expression; Figure S4: Effect of dexamethasone model on FOXO expression; Figure S5: Effect of dexamethasone model on myosin heavy chain mRNA expression; Figure S6: Effect of atrophy on glycolytic metabolism; Figure S7: Effect of dexamethasone on glycolytic metabolism; Table S1: Summary of qRT-PCR primers from Integrated DNA Technologies (Coralville, IA, USA); Table S2: Summary of primary antibodies used for western blot experiments.
