# Supplementary material for: The Effect of Dexamethasone-Mediated Atrophy on Mitochondrial Function and BCAA Metabolism During Insulin Resistance in C2C12 Myotubes
Source: Metabolites. 2025 May 13;15(5):322. doi: 10.3390/metabo15050322 (PMC12113135; doi:10.3390/metabo15050322)
Supplement: Supplementary file 1 [file metabolites-15-00322-s001.zip › metabolites-3440938-supplementary.pdf]

metabolites

MDPI

Supplemental Materials

The Effect of Dexamethasone-Mediated Atrophy on Mitochondrial Function and BCAA Metabolism During Insulin Resistance in C2C12 Myotubes

Kayla J. Ragland [1], Kipton B. Travis [1], Emmalie R. Spry [1], Toheed Zaman [2], Pamela M. Lundin [2] and Roger A. Vaughan [1,*]

[1] Department of Health and Human Performance, High Point University, High Point, NC 27268, USA; kragland@highpoint.edu (K.J.R.); ktravis@highpoint.edu (K.B.T.); espry@highpoint.edu (E.R.S.)
[2] Department of Chemistry, High Point University, High Point, NC 27268, USA; tzaman@highpoint.edu (T.Z.); plundin@highpoint.edu (P.M.L.)
* Correspondence: rvaughan@highpoint.edu

1. Complete Experimental Methods

1.1. Cell Culture

C2C12 mouse myoblasts from ATCC (Manassas, VA) were cultured in Dulbecco's Modified Eagle's Medium (DMEM) containing 4500mg/L glucose and supplemented with 20% heat-inactivated fetal bovine serum (FBS) and 100U/mL penicillin and 100μg/mL streptomycin in a humidified 5% CO_2 atmosphere at 37°C. Cells were grown to confluency with growth media changed every two to three days (using cell passages <20 for all experiments) as previously described [1]. Differentiation was accomplished by replacing growth media with DMEM supplemented with 2% horse serum and 100U/mL penicillin and 100μg/mL streptomycin for 6 days and insulin resistance was accomplished by the addition of insulin at 100nM for the final 3 days of differentiation as previously performed [2-6]. Similar to previous investigations [24-26,28], atrophy was induced by treating cells with either 1μM or 10μM dexamethasone (Santa Cruz Biotechnology) or DMSO control (final volume of DMSO was 0.1% vol:vol for all groups) for 24 hours. These concentrations of dexamethasone were selected as sufficient levels to induce atrophy [24-26,28], and because previous data in hepatocytes suggests that similar levels of dexamethasone alter BCKDH activity [38].

2.2. Quantitative Real Time Polymerase Chain Reaction (qRT-PCR)

Cells were differentiated and treated as described above. qRT-PCR was completed as previously described [1]. Briefly, total mRNA was extracted using the Trizol method and quantified (via NanoDrop from Thermo Fisher, Wilmington, DE), and cDNA was synthesized using the iScript cDNA Synthesis Kit from Bio-Rad (Hercules, CA) according to manufacturer's instructions. PCR primers were synthesized by Integrated DNA Technologies (Coralville, IA) (Table s1). Amplification of target genes were normalized to the housekeeping gene TATA binding protein (Tbp) which did not differ between groups (Fig s1). qRT-PCR reactions were performed using the CFX Connect System from Bio-Rad (Hercules, CA). SYBR Green-based PCR was performed using final primer concentrations at 3.75μM in a total volume of 10μl using per well. The following cycling parameters were used: 95˚C for 3 minutes followed by 40 cycles of 95˚C for 15 seconds, and 60˚C for 30

 https://doi.org/10.3390/metabo15050322

seconds. qRT-PCR reactions were performed using n=2 per treatment condition from 2 independent experiments with n=4 for the final analysis. Relative quantification was determined via ΔΔCt method.

2.3. Immunoblotting

Cells were differentiated and treated as described and collected on ice in RIPA and used for Western blots as previously described [1]. To assess insulin sensitivity, cells were treated as described above, followed by serum-free media stimulation with 100nM insulin for 30 minutes. Whole cell lysates were then prepared by harvesting the cells on ice in RIPA buffer supplemented with protease inhibitor, followed by incubation on ice for 60 minutes. Insoluble material was removed, and protein concentrations were determined by Bradford assay. Total protein (50µg or 100µg per sample) was size-separated by 10% sodium dodecyl sulfate polyacrylamide gel electrophoresis (SDS-PAGE) and electrotransferred to PVDF membranes. After blocking in TBST-5% non-fat milk powder for 1 hour, membranes were probed at 4ºC overnight with primary antibodies in TBST-5% non-fat milk powder (Table s2). Bound antibodies were detected by horseradish peroxidase-conjugated secondary antibodies from AbCam (Cambridge, MA) at a dilution of 1:5000 in TBST-5% non-fat milk powder for 1 hour at room temperature while shaking. Protein signal intensities were determined by chemiluminescence using the Clarity Western ECL substrate kit from Bio-Rad (Hercules, CA) and imaged using the ChemiDoc Touch from Bio-Rad (Hercules, CA). Relative signal intensities were quantified using Image Lab from Bio-Rad (Hercules, CA). Blots were performed using 2 replicates per condition performed across 2 independent experiments with n=4 for the final analysis.

2.4. Seahorse metabolic assays

Cells were seeded into Seahorse XFe96 culture plates, differentiated, and treated as described above, and assessed for metabolic capacity as previously performed [1]. Media was then replaced with XF Assay Media obtained from Agilent Technologies (Santa Clara, CA) containing glucose at 25mM, pyruvate at 1mM, and glutamine at 2mM. Following incubation, baseline measurements of oxygen consumption rate (OCR) and extracellular acidification rate (ECAR) were recorded as indicators of basal oxidative metabolism and glycolytic metabolism, respectively. Following basal measurements, each well was infused with oligomycin (an inhibitor of ATP synthase) at a final concentration of 2µM to induce maximal glycolytic metabolism. Cells were then exposed to carbonyl cyanide p-[trifluoromethoxy]-phenyl-hydrazone (FCCP) at 2µM to uncouple electron transport and induce peak OCR. Maximal respiration measurements were followed by the injection of rotenone at 1µM to reveal non-mitochondrial respiration. Basal and peak oxidative metabolism were normalized to non-mitochondrial OCR from each respective well. The Seahorse XFe96 Analyzer was run using a 6-minute cyclic protocol command (mix for 3 minutes and measure for 3 minutes). MitoStress assays included n=15-16 per group repeated with two independent experiments for n=30-32 per group for the final analysis. States of mitochondrial metabolism were calculated by subtracting non-mitochondrial respiration from basal or FCCP-induced peak mitochondrial oxygen consumption. Wells with negative OCR values or no response to injection were removed from the final analysis.

2.5. Fluorescent Staining and Fluorescent Microscopy

Immediately following the Seahorse metabolic assay described above, cells were fixed using 3.7% formaldehyde at 37°C with a 5% CO2 atmosphere. The fixing agent was then removed, and cells were stained with DAPI at 0.5µM in PBS and fluorescence was measured at 360/460nM which differed between groups (Fig s2). Cells were then stained

with 100μM nonyl acridine orange (NAO) (Fremont, CA) in PBS and incubated in the dark at room temperature for 10 minutes. Fluorescence was then measured using 485/525nm excitation/emission. Neutral lipid content was measured using Nile Red staining at 10μM PBS with 1% DMSO vol/vol using 530/645nm excitation/emission. All fluorescent measurements were made in triplicate and the average (less background) analyzed with n=15-16 per group repeated with two independent experiments with n=30-32 per group for the final analyses. Following fluorescent quantification, cells were imaged using the 10X objective using the Motic AE31E inverted microscope and Moticam Pro 252B (Causeway Bay, Hong Kong).

2.6. Liquid chromatography–mass spectrometry (LC–MS)

Extracellular BCAA content was assessed using parameters similar to previous experiments [12]. Chromatographic separation and quantification of leucine, isoleucine, and valine was performed using a Shimadzu Nexera UHPLC system equipped with a Phenomenex Kinetex C18 100Å column (100 X 3mm, 2.6μm) kept at a temperature of 30˚C connected to Shimadzu LCMS-8045 triple quadrupole mass spectrometer (Shimadzu, Kyoto, Japan) fitted with a DUIS ion source. The source used nebulizer gas 2.0L/minute, drying gas 10.0L/minute, desolvation line (DL) temperature 250˚C and heat block temperature 400˚C, with CID gas 230kPa. The mobile phases of A (water with 0.1% formic acid) and B (methanol 0.1% formic acid) were used at a flow rate of 0.4mL/min for the following gradient method: 0 minutes, 20% B; 1.7 minutes, 40% B; 5 minutes, 65% B; 8 minutes, 65% B; followed by 4 minutes 20% B for column equilibration. The injection volume was maintained at 1μL. This afforded reproducible retention time values for valine (0.415 minutes), isoleucine (0.521 minutes), and leucine (0.550 minutes).

Shimadzu LabSolution software version 5.97 was used to acquire and process the data. The fragmentation for each BCAA was optimized using MRM set to positive mode for valine (118.1 to 72.2 m/z, Q1 -23.0 V, CE -12.0 V, and Q3 -20.0 V), isoleucine (132.0 to 69.2 m/z, Q1 -10.0 V, CE -19.0 V, and Q3 -11.0 V), and leucine (132.1 to 43.2 m/z, Q1 -10.0 V, CE -26.0 V, and Q3 -18.0 V), with a dwell time of 100 msec.

A stock solution containing all BCAAs at a concentration of 8.0mM was obtained by dissolving each amino acid in water/methanol solution (50:50, v/v) and kept at 4˚C. Further dilutions with water/methanol were performed to assemble a calibration curve ranging from 3.125 to 100.0μM. Experiments were performed using 4 replicates per group for each of 2 independent experiments with n=8 for each group in the final analyses, with each sample measured in triplicate.

2.7. Statistical Analyses

Data are presented as dot plots with group means, or as group mean ± SE. Seahorse Mitostress data was initially analyzed using factorial ANOVA with time as a repeated measures factor and Bonferroni's correction for pairwise group differences. All other data were analyzed with two-way ANOVA with Bonferroni's correction for pairwise group differences. For small data set (n<24), data were analyzed using Prism 3.0. Larger data sets were analyzed using SPSS Version 29.0.0.0. Two-sided analyses were exclusively used, and values of $p < 0.05$ were used to identify significant differences between groups. Data were also assessed for normality using Kolmogorov-Smirnov's test and that data sets with non-normal data were analyzed by nonparametric post-hoc comparisons using Dunn's Multiple Comparison Test. In cases of suspected extreme outliers, Grubb's test was performed prior to the removal of any outlier. For clarity of sample size calculations, independent experiments indicate different versions of cells seeded and treated independently of other experiments, while independent biological sample/replicates are represented by independent wells within each experiment. Technical replicates represent multiple

measurements within a single sample or well, and averages of all technical replicates for each biological sample were used as n=1.

2. Supplementary Figures

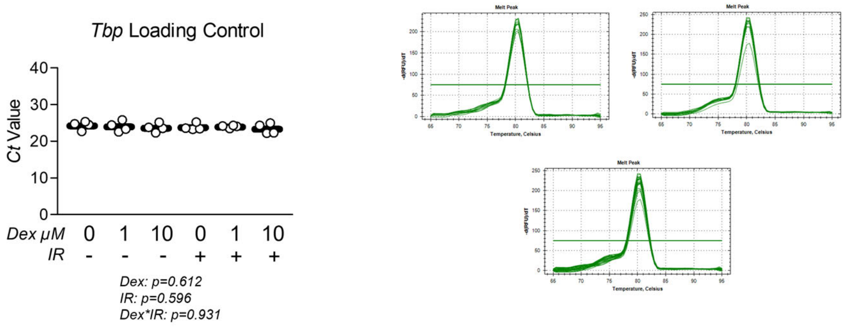

Figure S1. qRTPCR loading controls. Effect of treatment with dexamethasone (Dex) at 0µM, 1µM, or 10µM or DMSO control (0.1%) for 24 hours under insulin sensitive or insulin resistant (IR) conditions on tata binding protein (Tbp) expression with melt curves from 2 indepenent experiments (top) or merged (bottom). Notes: Two-way ANOVA with Bonferroni's corrections were used to analyze data. p values presented for main effects are listed for each outcome. Gene expression results revealed no significant effects. mRNA expression data are from 2 replicate wells from 2 independent experiments (n=4).

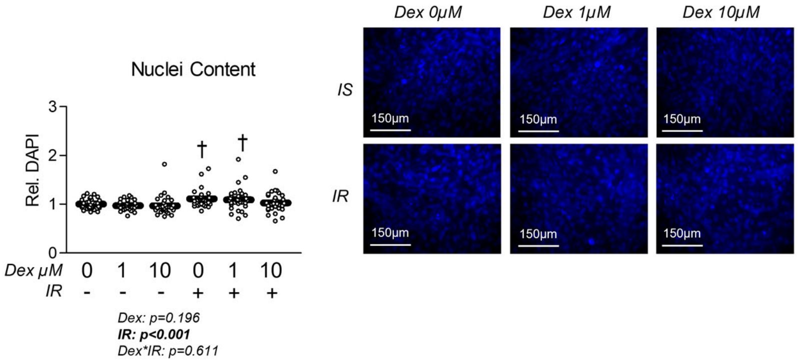

Figure S2. Nuclei content from the MitoStress Seahorse assays. Effect of dexamethasone (Dex) at 0µM, 1µM, or 10µM or DMSO control (0.1%) for 24 hours under insulin sensitive or insulin resistant (IR) conditions on nuclei content indicated by DAPI staining following Seahorse assays. Notes: Two-way ANOVA with Bonferroni's corrections were used to analyze data. p values presented for main effects are listed for each outcome. No significant differences between Dex treatments and respective true control within each level of insulin sensitivity was observed. † indicates p<0.05 between levels of insulin resistance within similar Dex concentrations. Images were captured using the 20X objective. Data are from 30-32 replicate wells from 2 independent experiments (n=30-32).

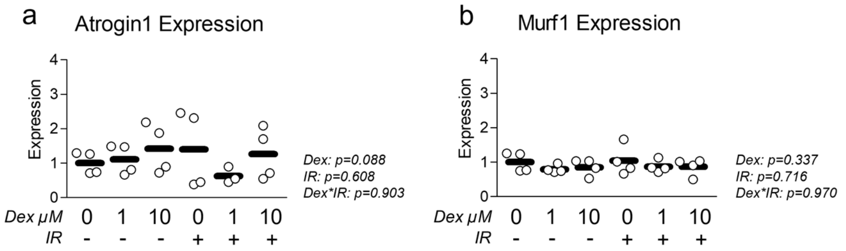

Figure S3. Atrophy-related gene expression. (a and b) Effect of treatment with dexamethasone (Dex) at 0μM, 1μM, or 10μM or DMSO control (0.1%) for 24 hours under insulin sensitive or insulin resistant (IR) conditions on (a) atrogin 1 and (b) Murf1 expression. Notes: Two-way ANOVA with Bonferroni's corrections were used to analyze data. p values presented for main effects are listed for each outcome. Gene expression results revealed no significant effects. mRNA expression data are from 2 replicate wells from 2 independent experiments (n=4).

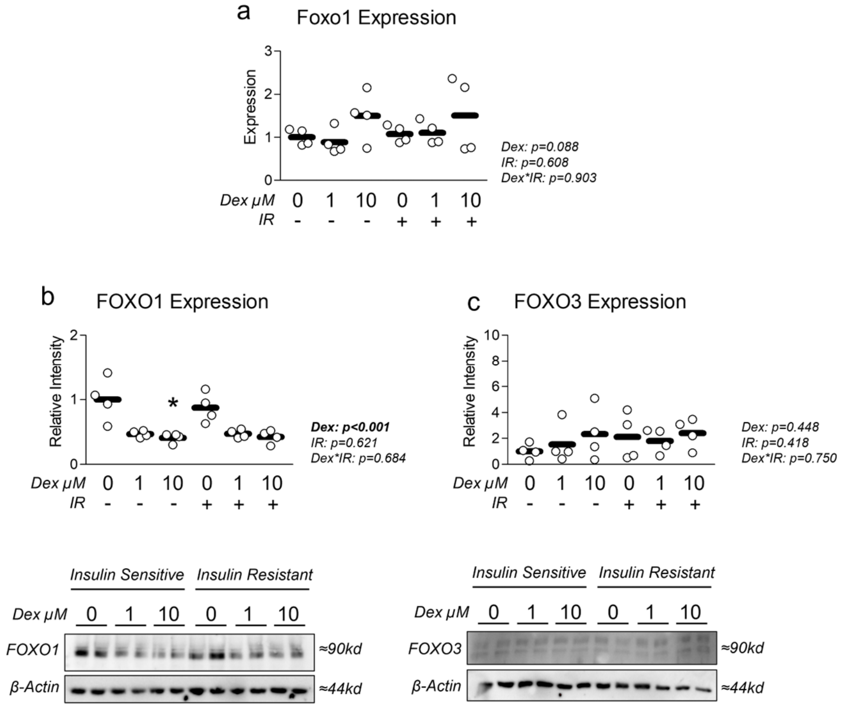

Figure S4. FOXO expression. (a) Effect of treatment with dexamethasone (Dex) at 0μM, 1μM, or 10μM or DMSO control (0.1%) for 24 hours under insulin sensitive or insulin resistant (IR) conditions on mRNA expression of Foxo1. (b and c) Effect of treatment described in "a" on protein expression of (b) Foxo1 and (c) FOXO3. Notes: Two-way ANOVA with Bonferroni's corrections were used to analyze data. p values presented for main effects are listed for each outcome. Gene expression results revealed no significant effects. * Indicates $p<0.05$ between Dex versus its true control within each level of insulin sensitivity. mRNA and protein expression data are from 2 replicate wells from 2 independent experiments (n=4).

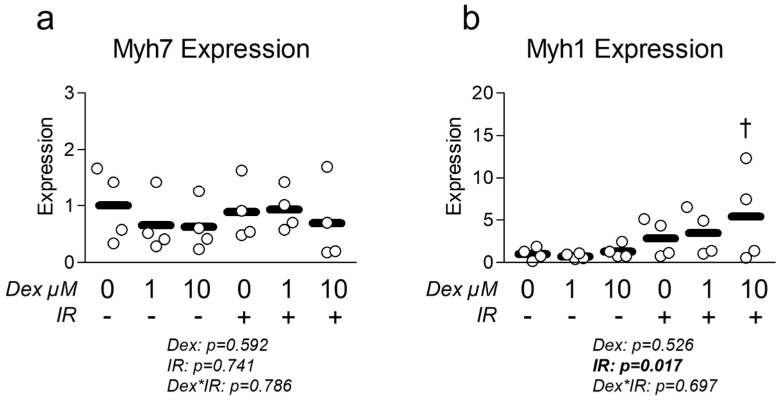

Figure S5. Myosin heavy chain gene expression. (a and b) Effect of treatment with dexamethasone (Dex) at 0μM, 1μM, or 10μM or DMSO control (0.1%) for 24 hours under insulin sensitive or insulin resistant (IR) conditions on (a) myosin heavy chain 7 (Myh7) and (b) myosin heavy chain 1 (Myh1) expression. Notes: Two-way ANOVA with Bonferroni's corrections were used to analyze data. p values presented for main effects are listed for each outcome. † indicates p<0.05 between levels of insulin resistance within similar Dex concentrations. No differences within each respective level of insulin resistance was observed. mRNA expression data are from 2 replicate wells from 2 independent experiments (n=4).

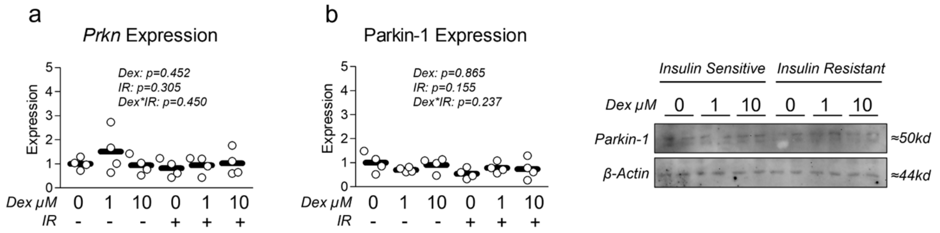

Figure S6 Parkin-1 expression. (a) Effect of treatment with dexamethasone (Dex) at 0μM, 1μM, or 10μM or DMSO control (0.1%) for 24 hours under insulin sensitive or insulin resistant (IR) conditions on mRNA expression of Parkin-1 (*Prkn*). (b) Effect of treatment described in "a" on protein expression of Parkin-1. Notes: Two-way ANOVA with Bonferroni's corrections were used to analyze data. p values presented for main effects are listed for each outcome. Gene expression results revealed no significant effects. * Indicates p<0.05 between Dex versus its true control within each level of insulin sensitivity. mRNA and protein expression data are from 2 replicate wells from 2 independent experiments (n=4).

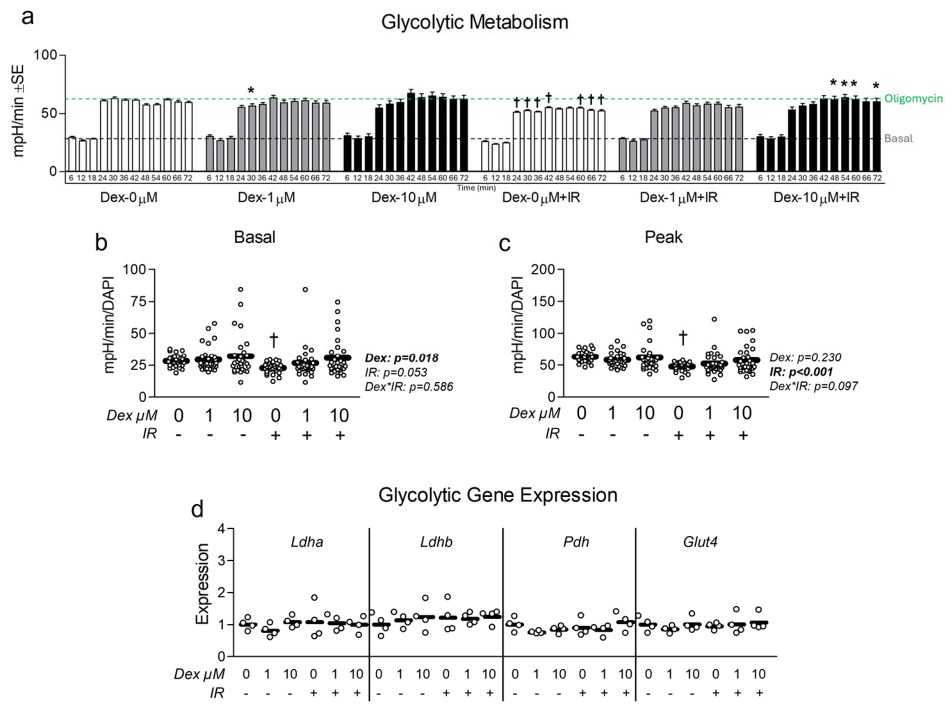

Figure S7. Effect of dexamethasone on glycolytic metabolism. (a) Extracellular acidification rate from MitoStress assay following treatment with dexamethasone (Dex) at 0μM, 1μM, or 10μM or DMSO control (0.1%) for 24 hours under insulin sensitive or insulin resistant (IR) conditions. (b and c) Effect of Dex on basal (b) and peak (c) glycolytic metabolism following normalization to cell nuclei content (presented in Fig s2). (d) Effect of Dex and/or IR on glycolytic mRNA expression. Notes: Panel "a" was analyzed using factorial ANOVA with time as a repeated measures factor and Bonferroni's correction for multiple comparisons. Two-way ANOVA with Bonferroni's corrections were used to analyze data. p values presented for main effects are listed for each outcome when significant main/interaction effects were observed. Gene expression results revealed no significant effects. * Indicates p<0.05 between Dex versus its true control within each level of insulin sensitivity. † indicates p<0.05 between levels of insulin resistance within similar Dex concentrations. Metabolism data are from 30-32 replicate wells from 2 independent experiments (n=30-32). mRNA expression data are from 2 replicate wells from 2 independent experiments (n=4).

3. Supplementary Tables

Table S1. Summary of qRT-PCR primers from Integrated DNA Technologies (Coralville, IA). Abbreviations: ATP synthase (Atp5o), branched-chain aminotransferase 1 (Bcat1), branched-chain aminotransferase 2 (Bcat2), branched-chain alpha-keto acid dehydrogenase (Bckdha), cytochrome C oxidase Subunit 5A (Cox5a), cytochrome B (cytb), dynamin-related protein 1 (Drp1), forkhead box protein O1 (Foxo1), glucose transporter 4 (Slc2a4 or Glut4), hydroxyisobutyrate dehydrogenase (Hibadh), lactate dehydrogenase a (Ldha), lactate dehydrogenase b (Ldhb), mitochondrial fission protein 1 (Fis1), mitofusin 2 (Mfn2), muscle RING-finger protein-1 (Murf1), myosin heavy chain 1 (Myh1), myosin heavy chain 7 (Myh7) nuclear respiratory factor 1 (Nrf1), parkin-1 (Prkn), peroxisome proliferator-activated receptor gamma coactivator 1 alpha (Ppargc1a), peroxisome proliferator-activated receptor gamma (Pparg), pyruvate dehydrogenase (Pdh), stearoyl-CoA desaturase (Scd1), sterol regulatory element-binding protein (Srebp1), TATA box binding protein (Tbp), and mitochondrial transcription factor A (Tfam).

Gene Abbreviation	Forward Sequence	Reverse Sequence
Atp5o	5'-AGGCCCTTTGCCAAGCTT-3'	5'-TTCTCCTTAGATGCAGCAGAGTACA-3'
Atrogin1	5'-GACTGGACTTCTCGACTG CC	5'-TCAGGGATGTGAGCTGTGAC
Bcat1	5′-CCCATCGTACCTCTTTCACCC-3′	5′-GGGAGCGTGGGAATACGTG-3′
Bcat2	5'-CGGACCCTTCATTCGTCAGA-3'	5'-CCATAGTTCCCCCCCAACTT-3'
Bckdha	5'-CCAGGGTTGGTGGGATGAG-3'	5'-GGCTTCCATGACCTTCTTTCG-3'
Cox5a	5'-GCTGCATCTGTGAAGAGGACAAC-3'	5'-CAGCTTGTAATGGGTTCCACAGT-3'
Cs	5'-TGAGAGGCATGAAGGGACTTGTGT-3'	5'-ATCTGTCCAGTTACCAGCAGCCAA-3'
Cytb	5'-ATATACACGCAAACGGAGCC-3'	5'-TAGGGCCGCGATAATAAATG-3'
Drp1	5'-TGCCTCAGATCGTCGTAGTG-3'	5'-TCTGGTGAAACGTGGACTAGC-3'
Fis1	5'-CAAAGAGGAACAGCGGGACT-3'	5'-CAACAGCCCTCGCACATACTT-3'
Foxo1	5'-GTACAGACAGTGGCAGGATTAG-3'	5'-GATGGACGGAATGAGAGGTAAA-3'
Slc2a4(Glut4)	5'-GATGAGAAACGGAAGTTGGAGAGA-3'	5'-GCACCACTGCGATGATCAGA-3'
Hibadh	5'- GCAGCGGTGTGTTCTAGGTC-3'	5'- ACACGTCATAGAGGATGAGTGG-3'
Ldha	5′-GGCTTGTGCCATCAGTATCT-3'	5′-CCCGCCTAAGGTTCTTCATTAT-3′
Ldhb	5'-AGTCTCCCGTGCATCCTCAA-3'	5'-AGGGTGTCCGCACTCTTCCT-3'
Mfn2	5'-TGATGTGGCCCAACTCCAAG-3'	5'-GTAACATCGATCCCAGGGCTGT-3'
Murf1	5'-GTGTGAGGTGCCTACTTGCTC-3'	5'-GCTCAGTCTTCTGTCCTTGGA-3'
Myh1	5'-ACCAAGGAGGAGGAACAGCAGC-3'	5'-GAATGCCTGTTTGCCCCTGGAG-3'
Myh7	5'-CAAGCAGCAGTTGGATGAGCGACT -3'	5'-TCCTCCAGCTCCTCGATGCGT-3'
Nrf1	5'-ACCCTCAGTCTCACGACTAT-3'	5'-GAACACTCCTCAGACCCTTAAC-3'
Prkn	5'-AAACCGGATGAGTGGTGAGT-3'	5'-AGCTACCGACGTGTCCTTGT-3'
Pdh	5'-GAAGGCCCTGCATTCAACTTC-3'	5'-ATAGGGACATCAGCACCAGTGA-3'
Ppargc1a	5'-GACAATCCCGAAGACACTACAG-3'	5'-AGAGAGGAGAGAGAGAGAGAGA-3'
Tbp	5'-GGGATTCAGGAAGACCACATA-3'	5'-CCTCACCAACTGTACCATCAG-3'
Tfam	5'-GAAGGGAATGGGAAAGGTAGAG-3'	5'-ACAGGACATGGAAAGCAGATTA-3'

Table S2. Summary of primary antibodies used for western blot experiments. Abbreviations: branched-chain aminotransferase 2 (BCAT2), branched-chain alpha-keto acid dehydrogenase E1*α* (BCKDHE1*α*), citrate synthase (CS), forkhead box protein O1A (FOXO1), forkhead box protein O3A (FOXO3), mouse monoclonal (MM), mechanistic target of rapamycin (mTOR), nuclear respiratory factor 1 (NRF1), peroxisome proliferator-activated receptor gamma coactivator 1 alpha (PGC-1*α*), and rabbit polyclonal (RP). Notes: Target molecular weight was based on product datasheet. Molecular weights for all targets were verified against sizes suggested by product brochures.

Protein Target	*Type*	*Dilution*	*Company*	*Item*	*Approx. Mol Wt.*	*Product Link*
pAkt (Ser 473)	RP	1:1000	SC Biotechnology	sc-7985-R	62kd	p-Akt1/2/3 (Ser 473)
Akt	RP	1:1000	Cell Signaling	9272	62kd	Akt Antibody#9272
β-Actin	RP	1:1000	SC Biotechnology	sc-130656	43kd	Datasheet
BCAT2	RP	1:1000	Bioss	BS-6589R	44kd	Datasheet
pBCKDHa (Ser 293)	RP	1:1000	AbCam	ab200577	50kd	Phospho BCKDHA (S293)
FOXO1	RP	1:1000	SC Biotechnology	sc-11350	80kd	FKHR (H-128): sc-11350
FOXO3	RP	1:1000	SC Biotechnology	sc-11351	87-99kd	FKHRL1 (H-144): sc-11351
mTOR	RP	1:1000	SC Biotechnology	sc-8319	211-245kd	sc-8319 - mTOR
pmTOR (Ser 2448)	RP	1:1000	SC Biotechnology	sc-101738	220kd	p-mTOR (Ser 2448)
NRF1	RP	1:1000	SC Biotechnology	sc-33771	68kd	Datasheet
Parkin-1	RP	1:1000	Cell Signaling	2132S	50kd	Parkin Antibody #2132
PGC-1α	RP	1:1000	SC Biotechnology	sc-13067	90kd	PGC-1α (H-300)
Total OXPHOS	MM	1:1000	AbCam	ab110413	Varied	Total OXPHOS

Additional Supporting References

[1] L.R. VanDerStad, E.C. Wyatt, R.A. Vaughan, The antidiabetic SGLT2 inhibitor canagliflozin reduces mitochondrial metabolism in a model of skeletal muscle insulin resistance, Diabet Med, 41 (2024) e15271.

[2] E.S. Lyon, M.E. Rivera, M.A. Johnson, K.L. Sunderland, R.A. Vaughan, Actions of chronic physiological 3-hydroxyisobuterate treatment on mitochondrial metabolism and insulin signaling in myotubes, Nutr Res, 66 (2019) 22-31.

[3] M.E. Rivera, E.S. Lyon, M.A. Johnson, K.L. Sunderland, R.A. Vaughan, Effect of valine on myotube insulin sensitivity and metabolism with and without insulin resistance., *Molecular and Cellular Biochemistry*, (2020).

[4] M.E. Rivera, E.S. Lyon, M.A. Johnson, R.A. Vaughan, Leucine increases mitochondrial metabolism and lipid content without altering insulin signaling in myotubes, Biochimie, 168 (2020) 124-133.

[5] N. Kumar, C.S. Dey, Metformin enhances insulin signalling in insulin-dependent and-independent pathways in insulin resistant muscle cells, Br J Pharmacol, 137 (2002) 329-336.

[6] N. Kumar, C.S. Dey, Development of insulin resistance and reversal by thiazolidinediones in C2C12 skeletal muscle cells, Biochem Pharmacol, 65 (2003) 249-257.

[7] H.J. Kim, S.W. Kim, S.H. Lee, D.W. Jung, D.R. Williams, Inhibiting 5-lipoxygenase prevents skeletal muscle atrophy by targeting organogenesis signalling and insulin-like growth factor-1, J Cachexia Sarcopenia Muscle, 13 (2022) 3062-3077.

[8] R. Kim, H. Kim, M. Im, S.K. Park, H.J. Han, S. An, J.S. Kang, S.J. Lee, G.U. Bae, BST204 Protects Dexamethasone-Induced Myotube Atrophy through the Upregulation of Myotube Formation and Mitochondrial Function, Int J Environ Res Public Health, 18 (2021).

[9] J.Y. Kim, H.M. Kim, J.H. Kim, J.H. Lee, K. Zhang, S. Guo, D.H. Lee, E.M. Gao, R.H. Son, S.M. Kim, C.Y. Kim, Preventive effects of the butanol fraction of, Heliyon, 8 (2022) e11597.

[10] N. Collao, P. Akohene-Mensah, J. Nallabelli, E.R. Binet, A. Askarian, J. Lloyd, G.M. Niemiro, J.W. Beals, S. van Vliet, R. Rajgara, A. Saleh, N. Wiper-Bergeron, S.A. Paluska, N.A. Burd, M. De Lisio, The role of L-type amino acid transporter 1 (Am J Physiol Cell Physiol, 323 (2022) C595-C605.

[11] A.G. Chicco, S.A. Adibi, W.Q. Liu, S.M. Morris, H.S. Paul, Regulation of gene expression of branched-chain keto acid dehydrogenase complex in primary cultured hepatocytes by dexamethasone and a cAMP analog, J Biol Chem, 269 (1994) 19427-19434.

[12] C.N. Rivera, M.M. Kamer, M.E. Rivera, R.M. Watne, T.C. Macgowan, A.J. Wommack, R.A. Vaughan, Insulin resistance promotes extracellular BCAA accumulation without altering LAT1 content, independent of prior BCAA treatment in a myotube model of skeletal muscle, Mol Cell Endocrinol, 559 (2023) 111800.

Disclaimer/Publisher's Note: The statements, opinions and data contained in all publications are solely those of the individual author(s) and contributor(s) and not of MDPI and/or the editor(s). MDPI and/or the editor(s) disclaim responsibility for any injury to people or property resulting from any ideas, methods, instructions or products referred to in the content.
